# The role of climate change anxiety in shaping childrearing intentions among people living in British Columbia

**DOI:** 10.3389/fpubh.2025.1642689

**Published:** 2025-09-11

**Authors:** Niloufar Aran, Aayush Sharma, Andreea Bratu, Kalysha Closson, Maya K. Gislason, Angel Kennedy, Carmen H. Logie, Jennifer L. Barkin, Robert S. Hogg, Kiffer G. Card

**Affiliations:** ^1^Faculty of Health Sciences, Simon Fraser University, Burnaby, BC, Canada; ^2^Factor-Inwentash Faculty of Social Work, University of Toronto, Toronto, ON, Canada; ^3^Community Medicine Department, Mercer University School of Medicine, Macon, GA, United States

**Keywords:** climate anxiety, childrearing, climate distress, political orientation, climate change, family planning

## Abstract

**Introduction:**

Climate change concerns have emerged as a factor in shaping childrearing intentions. Given extreme weather events, climate change-related anxiety has increased drastically in the region of British Columbia (BC), Canada. This study explored how worry about an increasingly uncertain future may be associated with people’s childrearing intentions in BC.

**Methods:**

This study used BC-CDMS (British Columbia Climate Distress Monitoring System) data from childless participants aged 16–44. We conducted multinomial logistic regression analyses (*n* = 441) to examine the association between climate change anxiety [measured using the Climate Change Anxiety Scale (CCAS)] and childrearing intentions. We controlled for covariates, including socio-demographic characteristics and generalized distress. A mediation analysis also tested whether political orientation mediates the primary relationship.

**Results:**

Participants who were undecided about having children (aOR = 1.58, 95% CI = 1.10–2.26) and those who planned not to have children (aOR = 1.64, 95% CI = 1.13–2.37) had higher CCAS scores compared to those who planned to have children. After controlling for covariates, climate change anxiety was still associated with childrearing intentions. Our mediation model indicated that political orientation scores partially mediate the relationship between climate anxiety and childrearing intentions.

**Discussion:**

Decision-makers should consider the impacts of climate anxiety and childrearing intentions on population and demographic shifts while supporting opportunities to reduce climate anxiety. Future research should consider the factors that influence and contribute to climate anxiety and climate-related distress, and their impact on childrearing intentions.

## 1 Introduction

Climate change concerns have emerged as an important factor in shaping childrearing intentions and family planning (1). Many popular media sources and opinion articles have well-documented this phenomenon (2–5). Recent research has provided empirical support to substantiate these claims (1), underscoring the need for more academic discourse on population decline (6). Climate researchers have contributed to this conversation with arguments that having fewer children is one of the most positively impactful environmental behaviors one can undertake (7), and many perspective pieces show that people are increasingly more conscious about having children given a progressively uncertain future due to the worsening impacts of climate change (8). Though this discourse has not always considered the views, preferences, and autonomy of would-be parents (especially mothers), policymakers have frequently identified population size as a viable intervention target to mitigate adverse human impacts on the environment in both scholarly outlets and the popular imagination (7, 9–18).

Indeed, in the World Scientists’ Warning of a Climate Emergency, academics from around the globe argued that “the world population must be stabilized – and, ideally, gradually reduced” (19). Over the last few decades, global fertility rates have declined in response to social and economic changes that have improved the status of women and children (20). Recent data now corroborate the hypothesis that these efforts influence people, as they increasingly choose to have children later in life and fewer children than 20 years ago (21). Choosing whether people want children and, if so, when to have them is an important decision shaped by core values and beliefs that are heavily influenced by the dynamic social discourse in which they exist (21). Reproductive autonomy is further shaped by multiple and overlapping systematic, cultural, political, and contextual forces (22–24). With this, some researchers have called the focus on overpopulation racist (25) and against feminism (26).

The reasons behind current decision-making trends regarding the number of children one has and when to have them are multifaceted and complex (8). According to Blackstone et al. (21), factors associated with the decision not to have children include gender, ethnicity, sexual orientation, political orientation, psychological distress, environmentalism, and feminism (21, 27). Other studies have also studied specific contributors to childrearing intentions, with significant differences found regarding gender and race (28); however, the effect of gender is non-significant in other studies (29). The impacts of climate change on childrearing intentions are also not equal, with individuals from lower socioeconomic backgrounds and those with less education being less likely to have children after experiencing extreme climate events (30). Thus, considering an intersectional lens can allow researchers to identify various forms of inequality and assess how these forms of inequality can operate together and exacerbate one another (31). An intersectional lens is also required to understand the differential and profound impacts of climate change and climate change anxiety and its impacts on childrearing intentions (30, 32).

Political orientation is a variable that many researchers are considering when examining attitudes and beliefs about the future, climate, and childrearing. Studies have shown that individuals with more conservative political affiliations tend to have lower levels of climate anxiety, a phenomenon observed in multiple countries, including the United States and Germany (33, 34). Additionally, studies indicate that political orientation influences childrearing intentions, with individuals with more liberal political affiliations exhibiting higher levels of climate reproductive concern (35), while those with more conservative political affiliations tend to have higher fertility intentions (36). Thus, understanding whether the potential relationship between climate change anxiety and childrearing intentions is affected by political orientation is a potential area of interest.

In the context of British Columbia (BC), Canada, there have been unprecedented extreme weather events, such as the extreme heat wave that took place in Western North America in the summer of 2021 and the disastrous flooding of early winter 2021 (37, 38). Prior research has shown that such events contribute to increased levels of climate change anxiety and distress (37), including concerns for the future. These concerns shape people’s perspectives as they navigate complex political and social spheres while simultaneously grappling with heightened levels of climate-related anxiety and distress (39–41).

Currently, few studies have examined climate anxiety in relation to other social and demographic factors that may influence childrearing intentions in the context of Canada and, more specifically, BC. The present study aims to address this current knowledge gap by examining the association between climate change anxiety and childrearing intentions within our sample population of adults in BC, Canada, in 2021–2022; we also aim to test whether this relationship is mediated by political orientation in order to assess whether anxiety about climate change might have an independent effect from broader political leanings.

## 2 Materials and methods

This analysis utilized cross-sectional survey data from the British Columbia Climate Distress Monitoring System (BC-CDMS). The CDMS was originally designed to explore how extreme weather events impacted British Columbians’ mental health using survey iterations before and after extreme climate events (37). The CDMS is described in greater detail, including recruitment strategies and power analysis, in previous literature (37). The CDMS recruited participants living in the province of BC, Canada, aged 16 years and above, between May and December 2021, in three iterations using paid social media advertisements on social media platforms Facebook and Instagram. The first survey wave was conducted between May 12th, 2021, and June 21st, 2021; wave two was conducted between July 15th, 2021, and July 18th, 2021 (after the 2021 Pacific Northwest American Heat Dome); and wave 3 was conducted between November 30th, 2021, and December 4th, 2021 (after the 2021 Pacific Northwest Atmospheric River and Flooding). BC-CDMS participants were screened for eligibility, provided informed consent, and completed a 10-min virtual questionnaire using the SurveyMonkey platform. This study’s sample was restricted to childless participants aged 16–44 years who had no missing data across the variables of interest.

### 2.1 Variables

The primary outcome of this study was participants’ childrearing intentions. The question in the survey was, “Do you have children?” The options in the survey were: (1) No, and I am not sure whether I want to have children; (2) No, and I do not plan on having children; (3) No, but I plan on having children one day (reference), and (4) Yes (which was removed from this study as we were looking at childless participants). The primary exposure variable in this study was the level of climate change anxiety measured by the Climate Change Anxiety Scale (CCAS) as a continuous variable (42). The CCAS (Cronbach’s alpha = 0.94) consists of 13 items assessing the frequency and persistence of anxious symptoms that emerge due to the negative impacts of climate change (e.g., “Thinking about climate change makes it difficult for me to concentrate,” “My concerns about climate change undermine my ability to work to my potential”). Each item is scored on a five-point Likert Scale ranging from “Never” to “Almost Always.” For each item, a higher score reflects a greater endorsement of the content covered by the item. Final scores are calculated as an average of scale items and range from 1 (Low Climate Change Anxiety) to 5 (High Climate Change Anxiety).

Confounders were selected using previous literature; selected confounders included: age (16–24 (reference), 25–44) (43), gender (man (reference), non-binary, woman) (43), ethnicity (White (reference), Chinese, Indigenous, South Asian, other) (44), sexual identity (heterosexual (reference), sexually diverse including asexual, bisexual, gay/lesbian, heteroflexible, pansexual, queer, questioning) (45), relationship status (in a relationship (reference), not in a relationship) (46), disability status (no (reference), yes) (46), income (less than $30,000 (reference), $30,000 to $59,999, $60,000 to $89,999, $90,000 or more) (47), education (high school or less (reference), Bachelor’s degree or higher, some post-secondary training) (47), geographic residence (urban (reference), rural, suburban) (48), and time spent on social media (less than 2 h (reference), 2 h or more) (49). Finally, we also included Kessler Psychological Distress Scale scores (K6) (50). The K6 consists of six items that measure the frequency and persistence of symptoms of non-specific psychological distress (e.g., “Felt restless,” “Felt Hopeless”), with a Cronbach’s alpha coefficient ranging from 0.89 to 0.92 (51). The Cronbach’s alpha coefficient for this sample was 0.89. Each item is scored on a five-point Likert Scale ranging from “None of the time” to “All of the time.” Final scores are calculated by summing the individual items and range from 0 (low non-specific psychological distress) to 24 (high non-specific psychological distress), which measures non-specific psychological distress using a 6-question 5-point Likert scale questionnaire (continuous) (52). For the mediation analysis, we assessed political orientation using a one-item, 7-point bipolar political orientation scale, ranging from extremely conservative to extremely liberal (53), with a continuous response (1–7) (54).

### 2.2 Study size

The total pooled sample size was 1704 participants. Of these, 946 were excluded because they had children, and 317 were excluded because of missing data on confounding variables. Thus, the final sample size for this study was 441 participants.

### 2.3 Analytical methods

All statistical analyses were conducted using SAS 9.4 and R version 4.1.2. We separated the values into three levels of the primary outcome variable (participants’ childrearing intentions). Frequencies and proportions are reported for categorical variables, while mean and standard deviation values are reported for continuous variables. We used a Chi-squared test for categorical variables, one-way ANOVA tests for continuous, normally distributed variables, and Kruskal–Wallis tests for continuous, non-normally distributed variables to test for differences between the variables.

We created minimally and fully adjusted multinomial logistic regression models to test the relationship between climate change anxiety and childrearing intentions. The multiple levels of the outcome variable were (1) being unsure about having children, (2) planning not to have children, and (3) planning to have children (reference). The minimally adjusted model controlled only for the design effects of time spent on social media and survey iteration. The fully adjusted model controlled for age, gender, ethnicity, sexual orientation, relationship status, disability status, income, education, geographic residence, time spent on social media, and non-specific psychological distress. A *p*-value of less than 0.05 was considered statistically significant.

Based on *a priori* knowledge and past literature on climate change anxiety and childrearing intentions, political orientation is a variable of unique interest (33–36). Thus, this study tested how political orientation impacted our minimally adjusted multinomial logistic regression model. We also developed a mediation model using a dichotomous outcome, comparing individuals who planned to have children with those who were unsure or planned not to have children, to examine the mediating effect of political orientation on the relationship between climate change anxiety and childrearing intentions. A *p*-value of less than 0.05 was considered statistically significant.

### 2.4 Ethics

The BC-CDMS was reviewed and approved by the Research Ethics Board at Simon Fraser University (SFU) (REB#: 30000309). Participants provided informed consent prior to study participation.

## 3 Results

Among the 441 participants who met the inclusion criteria, most were in the 25–44-year age group (63.7%) (Table 1), identified as cisgender women (48.3%) or non-binary (8.4%), and the majority identified as White (76.4%); 5.4 and 5.2% of the sample were Indigenous and Chinese, respectively. Most of our population also identified as heterosexual (58.5%); 41.5% identified as sexually diverse, while 51.9% had a Bachelor’s degree or higher. In total, 34.0% of participants planned to have children, 33.1% were unsure, and 32.9% planned not to have children.

**Table 1:** Sample description, stratified by childrearing intentions.

Descriptive characteristics	Overall	Plan to have children one day	Unsure about having children	Do not plan on having children	*x*^2^ *p*-value
	*n* = 441	*n* = 150	*n* = 146	*n* = 145	
	*n* (%)	*n* (%)	*n* (%)	*n* (%)	
Recruitment wave					
Wave 1	178 (40.4)	62 (41.3)	66 (45.2)	50 (34.5)	0.272
Wave 2	137 (31.1)	41 (27.3)	44 (30.1)	52 (35.9)	
Wave 3	126 (28.6)	47 (31.3)	36 (24.7)	43 (29.7)	
Age					
16–24	160 (36.3)	68 (45.3)	57 (39.0)	35 (24.1)	**0.001**
25–44	281 (63.7)	82 (54.7)	89 (61.0)	110 (75.9)	
Gender					
Man	191 (43.3)	80 (53.3)	54 (37.0)	57 (39.3)	**0.011**
Non-binary	37 (8.4)	8 (5.3)	11 (7.5)	18 (12.4)	
Woman	213 (48.3)	62 (41.3)	81 (55.5)	70 (48.3)	
Ethnicity					
White	337 (76.4)	107 (71.3)	104 (71.2)	126 (86.9)	**0.009**
Chinese	23 (5.2)	12 (8.0)	8 (5.5)	3 (2.1)	
Indigenous	24 (5.4)	10 (6.7)	6 (4.1)	8 (5.5)	
South Asian	16 (3.6)	7 (4.7)	8 (5.5)	1 (0.7)	
Other	41 (9.3)	14 (9.3)	20 (13.7)	7 (4.8)	
Sexual orientation					
Heterosexual	258 (58.5)	104 (69.3)	80 (54.8)	74 (51.0)	**0.003**
Sexually diverse	183 (41.5)	46 (30.7)	66 (45.2)	71 (49.0)	
Education					
High school or less	96 (21.8)	37 (24.7)	26 (17.8)	33 (22.8)	0.116
Some post-secondary training	116 (26.3)	44 (29.3)	31 (21.2)	41 (28.3)	
Bachelor’s degree or higher	229 (51.9)	69 (46.0)	89 (61.0)	71 (49.0)	
Relationship status					
In a relationship	292 (66.2)	102 (68.0)	92 (63.0)	98 (67.6)	0.605
Single	149 (33.8)	48 (32.0)	54 (37.0)	47 (32.4)	
Disability status					
No	377 (85.5)	131 (87.3)	134 (91.8)	112 (77.2)	**0.001**
Yes	64 (14.5)	19 (12.7)	12 (8.2)	33 (22.8)	
Income					
Less than $30,000	225 (51.0)	78 (52.0)	78 (53.4)	69 (47.6)	0.430
$30,000 to $59,999	115 (26.1)	33 (22.0)	43 (29.5)	39 (26.9)	
$60,000 to $89,999	67 (15.2)	26 (17.3)	18 (12.3)	23 (15.9)	
$90,000 or more	34 (7.7)	13 (8.7)	7 (4.8)	14 (9.7)	
Geographic residence					
Urban	208 (47.2)	68 (45.3)	72 (49.3)	68 (46.9)	0.100
Rural	164 (37.2)	54 (36.0)	47 (32.2)	63 (43.4)	
Suburban	69 (15.6)	28 (18.7)	27 (18.5)	14 (9.7)	
Time spent on social media					
Less than 2 h	183 (41.5)	62 (41.3)	62 (42.5)	59 (40.7)	0.953
2 h or more	258 (58.5)	88 (58.7)	84 (57.5)	86 (59.3)	
Climate change anxiety, per 1 point	2.06 (0.88)	1.79 (0.75)	2.17 (0.88)	2.23 (0.92)	**<0.001**
Kessler psychological distress, per 1 point	10.31 (5.74)	8.91 (5.86)	10.34 (5.58)	11.74 (5.44)	**<0.001**
Liberal political orientation, per	5.57 (1.55)	5.07 (1.74)	5.74 (1.39)	5.93 (1.35)	**<0.001**

Frequencies (N) and proportions (%) are reported for categorical variables; Means and standard deviations are reported for continuous Variables. *X*^2^ tests were used to test differences in categorical variables, one-way ANOVA tests were used for continuous normal variables, and Kruskal–Wallis tests were used for continuous non-normal variables. Sexually diverse indicates asexual, bisexual, gay/lesbian, heteroflexible, pansexual, queer, and questioning. Bolded values indicate statistical significance with a *p*-value less than or equal to 0.05.

Minimally adjusted models (Table 2), controlling only for design effects of time spent on social media and survey iteration, showed statistically significant higher CCAS scores for both those who were not sure if they wanted children (aOR = 1.79, 95% CI = 1.34–2.40) and those who did not want children (aOR = 1.89, 95% CI = 1.41–2.53). Including the effect of political orientation reduced these effects for both those who were not sure if they wanted children (aOR = 1.54, 95% CI = 1.14–2.09) and those who did not want children (aOR = 1.56, 95% CI = 1.15–2.11), but the association between CCAS and childrearing intentions was still statistically significant. The effect of political orientation on childrearing intentions was also statistically significant in individuals who were unsure about having children (aOR = 1.21, 95% CI = 1.02–1.42) and those who did not want children (aOR = 1.35, 95% CI = 1.13–1.60).

**Table 2:** Minimally adjusted and fully adjusted logistic regression associations between climate change anxiety with childrearing intentions (*n* = 441).

Primary exposure variable	Being unsure about having children		Not wanting children	
	Minimally adjusted odds ratio (95% CI)^a^	Fully adjusted odds ratio (95% CI)^b^	Minimally adjusted odds ratio (95% CI)^a^	Adjusted odds ratio (95% CI)^b^
Climate change anxiety	1.79 (1.34–2.40)	1.58 (1.10–2.26)	1.89 (1.41–2.53)	1.64 (1.13–2.37)

a Adjusted for time spent on social media and survey iteration.

b Adjusted for age, gender, ethnicity, sexual orientation, relationship status, disability status, income, education, geographic residence, time spent on social media, and non-specific psychological distress.

Variance inflation factors of a linearized model were calculated to test for multicollinearity in our final model. All factors had acceptable values, indicating no multicollinearity. A Hosmer-Lemeshow test was also conducted on our final model and indicated an acceptable model fit (X^2^ = 12.705, df = 16, p-value = 0.6942). McFadden’s Pseudo *R*^2^ also indicated an acceptable fit, with a value of 0.1156 on the multinomial model.

In the fully adjusted multinomial model (Table 2), adjusting for age, gender, ethnicity, sexual orientation, relationship status, disability status, income, education, geographic residence, time spent on social media, and non-specific psychological distress, participants who were undecided about having children had higher CCAS scores (aOR = 1.58, 95% CI = 1.10–2.26) and those who planned on not having children were older (25–44, aOR = 3.94, 95% CI = 2.05–7.57) and had higher CCAS scores (aOR = 1.64, 95% CI = 1.13–2.37).

**Figure 1:**
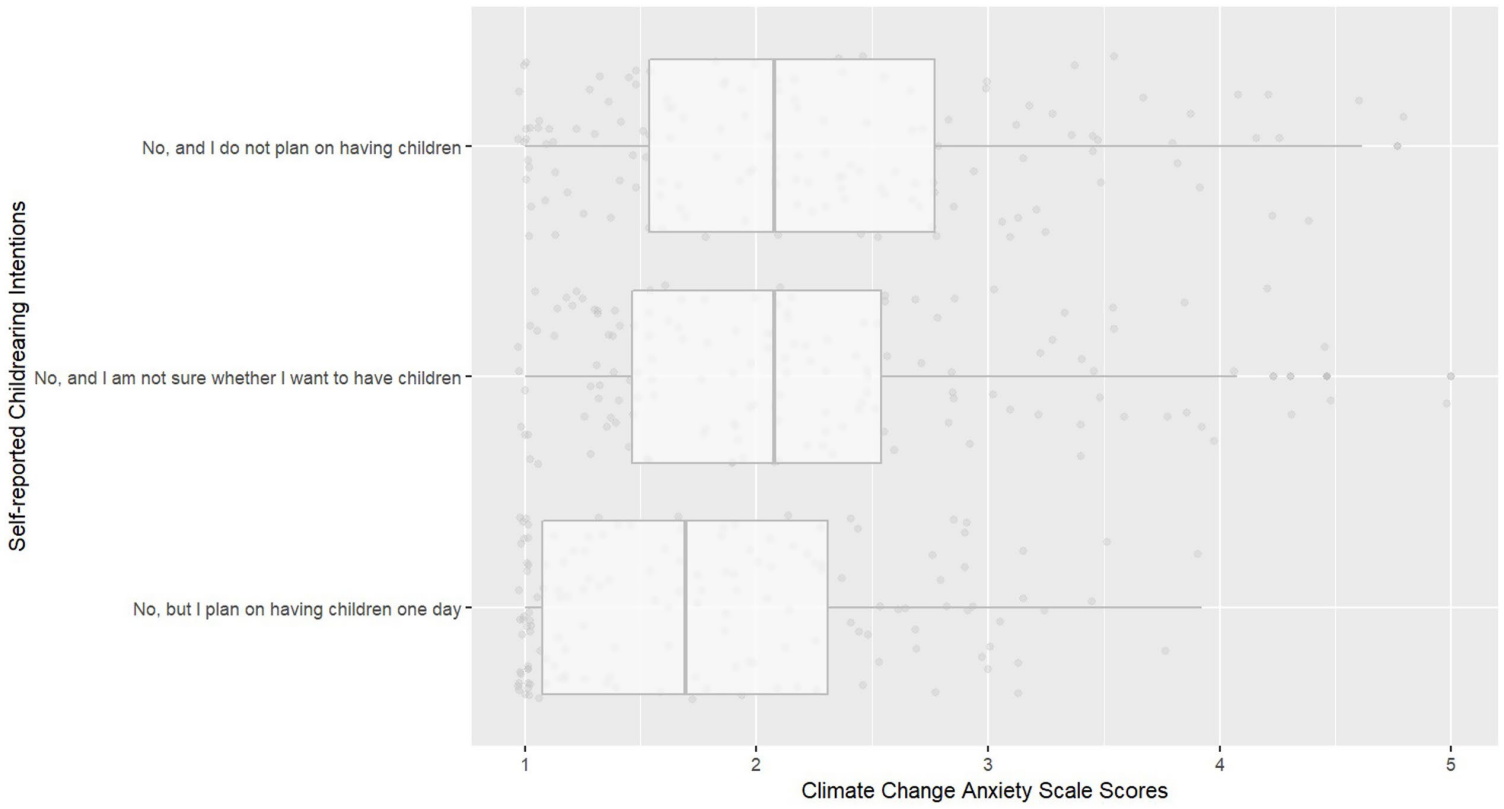
Boxplots for climate change anxiety scale scores, stratified by self-reported childrearing intentions.

**Figure 2:**
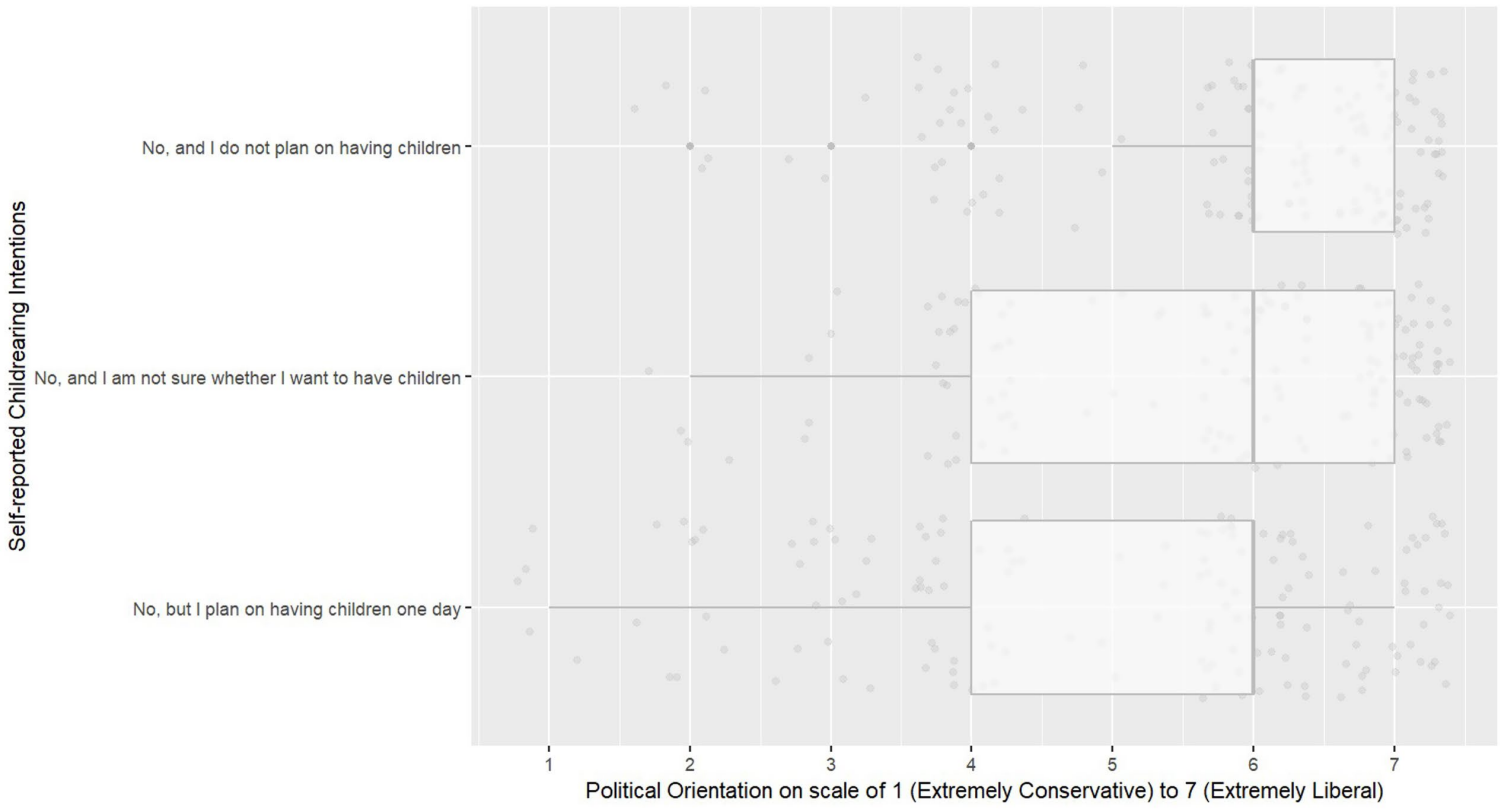
Boxplots for political orientation scores, stratified by self-reported childrearing intentions.

Figures 1, 2 illustrate the differences in CCAS scores and political orientation scores based on whether participants planned to have children, were unsure about having children, or planned not to have children.

The mediation analysis (Table 3) revealed that the effect of political orientation mediated 25.5% of the effect of CCAS scores on childrearing intentions, which was statistically significant with a *p*-value of less than 0.002.

**Table 3:** Mediation model of the effect of political orientation as a mediator on the relationship between childrearing decision-making and climate change anxiety (*n* = 441).

Mediation outcome types	Estimate	Lower bound	Upper bound	*p*-value
Indirect effect	−0.0359	−0.0567	−0.01	0.002**
Direct effect	−0.1049	−0.1595	−0.05	<0.0002**
% Mediated	0.2549	0.0992	0.46	0.002**

Mediation effects are the average effects between the “control” and “treatment” categories for the outcome variable. The outcome variable has been dichotomized [planning to have children (control) and unsure/not planning on having children (treatment)]. **Indicates statistical significance with a *p*-value less than or equal to 0.01.

## 4 Discussion

We found that participants who indicated that they were unsure about having children and those who did not plan on having children had higher CCAS scores, which revealed higher levels of climate-related anxiety. These findings remained significant in the fully adjusted analysis, where we controlled for age, gender, ethnicity, sexual orientation, relationship status, disability status, income, education, geographic residence, time spent on social media, and non-specific psychological distress. Our mediation analysis showed that political orientation scores mediated the effect of climate change anxiety on childrearing intentions. This finding suggests that part of the relationship between climate change anxiety and childrearing intentions is mediated through political orientation, representing an indirect effect. Importantly, we found that the direct effect of climate change anxiety on childrearing intentions was statistically significant.

The present findings align with several studies done in other contexts. Our study found that participants in Canada who indicated they did not plan on having children had higher levels of climate-related anxiety, with this seen in minimally and fully adjusted multinomial models. Previous studies have reported similar findings, suggesting a potential link between climate emotions and childrearing intentions. For example, a study by Schneider-Mayerson and Leong (1) found that, in a sample of 607 Americans aged 27–44, the majority (~60%) were worried about the carbon footprint that bringing kids into the world will have, while the vast majority (~96%) were concerned about the well-being of their current or future children in a world impacted by climate change. Another survey in America by Helm et al. (35) found that individuals with high climate reproductive concerns were less likely to want children, but this did not limit their desire to have only one child; the authors hypothesized that having one child could be a way to remain climate-conscious while being environmentally child-free. Another article by Fu et al. (55) reported a similar finding, where 173 young, educated, and climate-conscious individuals in China expressed deep concern about how climate change would impact their potential children. However, climate change did not rank highly among the factors influencing these participants’ childrearing intentions (55).

Concerning political orientation and climate distress, our study found that those with more liberal political orientation scores had higher levels of climate anxiety, with this being statistically significant in those who were unsure about having children and those who did not want children. Our study also found that the relationship between climate change anxiety and childrearing intentions was mediated by political orientation. A study conducted in several European countries similarly found that individuals who positioned themselves further to the right on the political spectrum were significantly less concerned about climate change (56). Additionally, in the United States, McCright and colleagues have found that Liberals and Democrats are more likely to express personal concern about climate change and recognize the human influence on this global problem than conservatives and Republicans (57). At a population level, we see that those with more liberal political orientations of reproductive age are displaying increasingly high levels of climate anxiety and having higher levels of climate reproductive concern (35). However, it is also possible that climate change anxiety could impact one’s political orientation rather than the other way around, with the directionality of this association unclear.

Climate change is a population-level concern that could contribute to demographic shifts and changes in population, particularly in light of trends regarding population decline and the growing discourse around having fewer children (6, 7). This effect could also intensify over time, given the more frequent and worsening climate events that have been predicted and the increasing awareness and concern about climate change among the public (58). However, it is also possible that people may face issue fatigue regarding climate change and become disengaged over time, with discussions about it decreasing and fewer climate-friendly solutions being adopted (59). Implementing pro-environmental behaviors and educating people on the benefits of decreasing their carbon footprint could, in turn, effectively alleviate their climate anxiety while simultaneously contributing to habits that will decrease our carbon footprint and create a sustainable future (60). Another factor not discussed in this article is climate-related litigation, as seen in the UN Environment Programme Global Climate Litigation Report, which may impact climate-related anxiety in either positive or negative ways, similar to the mechanisms of issue fatigue or increased awareness (59–61).

Fundamentally, many factors influence childrearing intentions and family planning, including individual choice and societal pressures. However, when a global phenomenon like climate change brings forward feelings of anxiety and distress while contributing to people fearing bringing children into the world and childrearing intentions worldwide, work must be done to understand this problem better. We emphasize the importance of addressing climate change on a global scale and the need for individual-level mitigation strategies to alleviate climate-related distress and anxiety, ultimately helping build a more sustainable future for future generations.

### 4.1 Strengths and limitations

Our survey engaged a sample of British Columbians across three waves of data collection, and a significant strength of our study is the timeliness of the data collection following the occurrence of heat waves in the province of BC (37). The opportunity to collect real-life and real-time evidence to generate knowledge significantly increases the ecological validity of our study while substantially reducing recall bias. However, our study is not without limitations. We utilized an online convenience sample, which introduces the possibility of non-response. We employed multinomial methods and adjusted for potential confounding effects, although there may be variables not accounted for in our analyses. As the data gathered was through a short online survey, we were unable to include extensive measures of climate distress. The political orientation variable was a one-question variable and, therefore, a simplistic measure of political orientation. Another limitation is that the measure used in this study was gender. Sex-assigned at birth would be a better variable, as the implications of this study differ depending on whether an individual was born with a uterus or not. However, the CDMS did not ask a question concerning sex-assigned at birth. Thus, future studies could assess whether climate change anxiety affects childrearing intentions using sex-assigned at birth. We also recognize that the CCAS scale will require ongoing validation and comparison with other climate anxiety scales. Another limitation is that we do not know the directionality of the effect observed in this study for the regression or mitigation analyses. Due to the study design, we cannot definitively determine whether climate change anxiety influences childrearing intentions or vice versa. Future studies could employ longitudinal research to assess this phenomenon better.

### 4.2 Suggestions for future research

While this analysis successfully identified the aforementioned associations, we were unable to thoroughly examine the multitude of complex and intersecting reasons and influences that may lead an individual to decide whether or not to have children. We recommend conducting more qualitative research in this field, particularly considering intersectionality and efforts to understand the pathways by which people choose not to have children, as well as the role of climate change in their decision-making. Additionally, future research could examine the associations between climate change anxiety and childrearing intentions with a larger sample size and over a larger geographic area. An interesting future research study could also investigate whether climate anxiety remains at the same level well after a climate disaster has occurred and memories have faded, with follow-up questions testing how time impacts childrearing desires, intentions, and planning change.

## 5 Conclusion

This study found that those with higher levels of climate anxiety were less likely to have children or were unsure about having children, with this effect independent of one’s socio-demographic background or lived experiences of psychological distress. This study also found that political orientation mediated this effect. Given the escalating rates of climate change and increasing climate-related anxiety, decision-makers should consider the impacts of climate anxiety and childrearing intentions on population and demographic shifts. Efforts to understand the complex relationship between climate-related anxiety and other social and environmental factors that shape people’s childrearing intentions require further investigation, given increasingly common extreme weather events and elevated levels of climate anxiety.

## Data Availability

The datasets presented in this study can be found in online repositories. The names of the repository/repositories and accession number(s) can be found at: https://mhcca.ca/datasets - MHCCA Data Holdings.

## References

[1] Schneider-Mayerson M Leong KL. Eco-reproductive concerns in the age of climate change. Clim Chang. (2020) 163:1007–23. doi: 10.1007/s10584-020-02923-y

[2] Carrington D.. Want to fight climate change? Have fewer children. Guardian. (2017); Available online at: https://www.theguardian.com/environment/2017/jul/12/want-to-fight-climate-change-have-fewer-children

[3] Clifford C. (2023). 53% of parents say climate change affects their decision to have more kids. CNBC. Available online at: https://www.cnbc.com/2023/06/20/climate-change-affects-53percent-of-parents-decision-to-have-more-kids.html

[4] Cottrell S. (2023). Is climate change becoming a factor in family planning? A new survey says yes. Parents. Available online at: https://www.parents.com/climate-change-affects-parents-and-kids-7558115

[5] Wray B. (2022). Deciding to have a baby amid the climate crisis: whatever you’re feeling, you’re not alone. CBC Documentaries. Available online at: https://www.cbc.ca/documentaries/deciding-to-have-a-baby-amid-the-climate-crisis-whatever-you-re-feeling-you-re-not-alone-1.6662734

[6] Coole D. Too many bodies? The return and disavowal of the population question. Environ. Politics. (2013) 22:195–215. doi: 10.1080/09644016.2012.730268

[7] Wynes S Nicholas KA. The climate mitigation gap: education and government recommendations miss the most effective individual actions. Environ Res Lett. (2017);12. Available online at: https://www.proquest.com/docview/2549210763/abstract/3B4B67BDB4C7465CPQ/1

[8] Bielski Z.. (2019). The new ‘childfree’: fearful amid climate change, some young Canadians abandon plans to have children. The Globe and Mail. Available online at: https://www.theglobeandmail.com/canada/article-the-new-childfree-fearful-amid-climate-change-some-young-canadians/ (Accessed March 21, 2025)

[9] Center for Biological Diversity. Population pressure and the climate crisis. Available online at: https://www.biologicaldiversity.org/programs/population_and_sustainability/climate/# (Accessed March 25, 2025).

[10] Crist E Mora C Engelman R. The interaction of human population, food production, and biodiversity protection. Science. (2017) 356:260–4. doi: 10.1126/science.aal2011, PMID: 28428391

[11] Hickey C Rieder TN Earl J. Population engineering and the fight against climate change: social theory and practice. Soc Theory Pract. (2016) 42:845–70. doi: 10.5840/soctheorpract2016424

[12] Holmes SJ. The control of population growth. Science. (1937) 86:181–7. doi: 10.1126/science.86.2226.181, PMID: 17812806

[13] Jaffe AJ. Obstacles to population control. Science. (1968) 159:481–1. doi: 10.1126/science.159.3814.481.a, PMID: 5635147

[14] O’Neill BC Dalton M Fuchs R Jiang L Pachauri S Zigova K. Global demographic trends and future carbon emissions. Proc Natl Acad Sci USA. (2010) 107:17521–6. doi: 10.1073/pnas.1004581107 20937861 PMC2955139

[15] O’Neill BC Liddle B Jiang L Smith KR Pachauri S Dalton M. Demographic change and carbon dioxide emissions. Lancet. (2012) 380:157–64. doi: 10.1016/S0140-6736(12)60958-1 22784534

[16] Population Matters. Climate change. Available online at: https://populationmatters.org/climate-change/ (Accessed March 25, 2025).

[17] Sax K. World population: control or crisis? Science. (1969) 163:763–4. doi: 10.1126/science.163.3869.763, PMID: 17807976

[18] Teitelbaum M. Population control. Science. (1975) 190:11–1. doi: 10.1126/science.190.4209.11

[19] Ripple WJ Wolf C Newsome TM Barnard P Moomaw WR. World scientists’ warning of a climate emergency. Bioscience. (2020) 70:8–12. doi: 10.1093/biosci/biac083

[20] Kebede E Goujon A Lutz W. Stalls in Africa’s fertility decline partly result from disruptions in female education. Proc Natl Acad Sci USA. (2019) 116:2891–6. doi: 10.1073/pnas.1717288116, PMID: 30718411 PMC6386713

[21] Blackstone A Stewart MD. Choosing to be childfree: research on the decision not to parent. Sociol Compass. (2012) 6:718–27. doi: 10.1111/j.1751-9020.2012.00496.x

[22] Brown BB. Facing the “black peril”: the politics of population control in South Africa. J South Afr Stud. (1987) 13:256–73. doi: 10.1080/03057078708708144, PMID: 11617501

[23] Kuumba MB. Perpetuating neo-colonialism through population control: South Africa and the United States. Afr Today. (1993) 40:79–85. 12286951

[24] Morgan LM. Reproductive governance, redux. Med Anthropol. (2019) 38:113–7. doi: 10.1080/01459740.2018.1555829, PMID: 30848982

[25] Lee YH. Not just against overpopulation: White supremacy in population control initiatives. Sociol Compass. (2025) 19:e70034. doi: 10.1111/soc4.70034

[26] Hendrixson A Ojeda D Sasser JS Nadimpally S Foley EE Bhatia R. Confronting populationism: feminist challenges to population control in an era of climate change. Gend Place Cult. (2020) 27:307–15. doi: 10.1080/0966369X.2019.1639634

[27] Blackstone A. Childfree by choice: the movement redefining family and creating a new age of Independence. New York, NY: Penguin Publishing Group (2019).

[28] Sasser JS Merchant EK. Climate emotions, parenting plans, and racial difference in the United States. J Clim Chang Health. (2024) 19:100346. doi: 10.1016/j.joclim.2024.100346 PMC12851343 41647319

[29] Zimmermann G Darwiche J Messerli-Bürgy N Van Petegem S Mouton B Venard G. “Bringing children in a burning world?” the role of climate anxiety and threat perceptions in childbearing motivations of emerging adults in Switzerland. Emerg Adulthood. (2024) 12:925–38. doi: 10.1177/21676968241258270

[30] Sellers S Gray C. Climate shocks constrain human fertility in Indonesia. World Dev. (2019) 117:357–69. doi: 10.1016/j.worlddev.2019.02.003, PMID: 31213734 PMC6581515

[31] Crenshaw K. On intersectionality: essential writings. New York: New Press (2019).

[32] Kaijser A Kronsell A. Climate change through the lens of intersectionality. Environ Politics. (2014) 23:417–33. doi: 10.1080/09644016.2013.835203

[33] Knollenborg L Sommer S. Diverging beliefs on climate change and climate policy: the role of political orientation. Environ Resour Econ. (2023) 84:1031–49. doi: 10.1007/s10640-022-00747-1

[34] Ziegler A. Political orientation, environmental values, and climate change beliefs and attitudes: an empirical cross country analysis. Energy Econ. (2017) 63:144–53. doi: 10.1016/j.eneco.2017.01.022

[35] Helm S Kemper J White S Dean D. Exploring climate-reproductive concern: factors influencing hesitancy towards parenthood in the context of the climate crisis. Environ Sociol. (2024) 11:1–15. doi: 10.1080/23251042.2024.2408779

[36] Arpino B Mogi R. Is intending to have children rightist? A research note on political ideology and fertility intentions. Stat Politics Policy. (2024) 15:117–36. doi: 10.1515/spp-2023-0038

[37] Bratu A Card KG Closson K Aran N Marshall C Clayton S. The 2021 Western north American heat dome increased climate change anxiety among British Columbians: results from a natural experiment. J Clim Chang Health. (2022) 6:100116. doi: 10.1016/j.joclim.2022.100116

[38] Richards-Thomas TS Déry SJ Stewart RE Thériault JM. Climatological context of the mid-November 2021 floods in the province of British Columbia, Canada. Weather Clim Extremes. (2024) 45:100705. doi: 10.1016/j.wace.2024.100705

[39] Gislason MK Kennedy AM Witham SM. The interplay between social and ecological determinants of mental health for children and youth in the climate crisis. Int J Environ Res Public Health. (2021) 18:4573. doi: 10.3390/ijerph18094573, PMID: 33925907 PMC8123462

[40] Hickman C Marks E Pihkala P Clayton S Lewandowski RE Mayall EE. Climate anxiety in children and young people and their beliefs about government responses to climate change: a global survey. Lancet Planet Health. (2021) 5:e863–73. doi: 10.1016/S2542-5196(21)00278-3, PMID: 34895496

[41] Léger-Goodes T Malboeuf-Hurtubise C Mastine T Généreux M Paradis PO Camden C. Eco-anxiety in children: a scoping review of the mental health impacts of the awareness of climate change. Front Psychol. (2022) 13:872544. doi: 10.3389/fpsyg.2022.872544, PMID: 35959069 PMC9359205

[42] Clayton S Karazsia BT. Development and validation of a measure of climate change anxiety. J Environ Psychol. (2020) 69:101434. doi: 10.1016/j.jenvp.2020.101434

[43] Latkin CA Dayton L Lee DI Yi G Uzzi M. Correlates of levels of willingness to engage in climate change actions in the United States. Int J Environ Res Public Health. (2021) 18:9204. doi: 10.3390/ijerph18179204, PMID: 34501794 PMC8431161

[44] Schuldt JP Pearson AR. The role of race and ethnicity in climate change polarization: evidence from a U.S. national survey experiment. Clim Chang. (2016) 136:495–505. doi: 10.1007/s10584-016-1631-3, PMID: 40873743

[45] Alibudbud R. Gender in climate change: safeguarding LGBTQ+ mental health in the Philippine climate change response from a minority stress perspective. J Prev Med Public Health. (2023) 56:196–9. doi: 10.3961/jpmph.22.501, PMID: 37055362 PMC10111101

[46] Gaskin CJ Taylor D Kinnear S Mann J Hillman W Moran M. Factors associated with the climate change vulnerability and the adaptive capacity of people with disability: a systematic review. Weather Clim Soc. (2017) 9:801–14. doi: 10.1175/WCAS-D-16-0126.1

[47] van der Linden S. The social-psychological determinants of climate change risk perceptions: towards a comprehensive model. J Environ Psychol. (2015) 41:112–24. doi: 10.1016/j.jenvp.2014.11.012

[48] Ren H Wu X Zhao W Cheng J. Potential risks of PM2.5 in urban and suburban environments: a dual perspective on chemical constituents and pollution sources. Aerosol Air Qual Res. (2024) 24:230315. doi: 10.4209/aaqr.230315

[49] Mavrodieva AV Rachman OK Harahap VB Shaw R. Role of social media as a soft power tool in raising public awareness and engagement in addressing climate change. Climate. (2019) 7:122. doi: 10.3390/cli7100122

[50] Brosch T. Affect and emotions as drivers of climate change perception and action: a review. Curr Opin Behav Sci. (2021) 42:15–21. doi: 10.1016/j.cobeha.2021.02.001

[51] Easton SD Safadi NS Wang Y Hasson RG. The kessler psychological distress scale: translation and validation of an Arabic version. Health Qual Life Outcomes. (2017) 15:215. doi: 10.1186/s12955-017-0783-9, PMID: 29078774 PMC5658946

[52] Kessler RC Andrews G Colpe LJ Hiripi E Mroczek DK Normand SLT. Short screening scales to monitor population prevalences and trends in non-specific psychological distress. Psychol Med. (2002) 32:959–76. doi: 10.1017/S0033291702006074, PMID: 12214795

[53] Tobler C Visschers VHM Siegrist M. Addressing climate change: determinants of consumers’ willingness to act and to support policy measures. J Environ Psychol. (2012) 32:197–207. doi: 10.1016/j.jenvp.2012.02.001

[54] Jost JT. The end of the end of ideology. Am Psychol. (2006) 61:651–70. doi: 10.1037/0003-066X.61.7.651, PMID: 17032067

[55] Fu X Schneider-Mayerson M Montefrio MJF. The reproductive climate concerns of young, educated Chinese: ‘when the nest is upset, no egg is left intact.’. Environ Sociol. (2023) 9:200–15. doi: 10.1080/23251042.2022.2132629

[56] Gregersen T Doran R Böhm G Tvinnereim E Poortinga W. Political orientation moderates the relationship between climate change beliefs and worry about climate change. Front Psychol. (2020) 11:1573. doi: 10.3389/fpsyg.2020.01573/full 32765360 PMC7378799

[57] McCright AM Dunlap RE. The politicization of climate change and polarization in the American public’s views of global warming, 2001–2010. Sociol Q. (2011) 52:155–94. doi: 10.1111/j.1533-8525.2011.01198.x

[58] Yusa A Berry P Cheng JJ Ogden N Bonsal B Stewart R. Climate change, drought and human health in Canada. Int J Environ Res Public Health. (2015) 12:8359–412. doi: 10.3390/ijerph120708359, PMID: 26193300 PMC4515727

[59] Morrison M Parton K Hine DW. Increasing belief but issue fatigue: changes in Australian household climate change segments between 2011 and 2016. PLoS One. (2018) 13:e0197988. doi: 10.1371/journal.pone.0197988, PMID: 29912888 PMC6005570

[60] Kapeller ML Jäger G. Threat and anxiety in the climate debate—an agent-based model to investigate climate scepticism and pro-environmental behaviour. Sustainability. (2020) 12:1823. doi: 10.3390/su12051823

[61] UN Environment Programme UNEP. (2023). Global climate litigation report: 2023 status review. Available online at: https://www.unep.org/resources/report/global-climate-litigation-report-2023-status-review (Accessed April 22, 2025).

