# Supplementary material for: The role of climate change anxiety in shaping childrearing intentions among people living in British Columbia
Source: Front Public Health. 2025 Sep 11;13:1642689. doi: 10.3389/fpubh.2025.1642689 (PMC12460336; doi:10.3389/fpubh.2025.1642689)
Supplement: Supplementary file 1 [file Data_Sheet_1.pdf]

Climate Change Impacts Survey

Informed Consent

Principal Investigators
Dr. Robert S. Hogg, Professor, Faculty of Health Sciences, Simon Fraser University (rhogg@sfu.ca)
Dr. Kiffer Card, Adjunct Professor, Simon Fraser University (kcard@sfu.ca)

Introduction
You are being asked to participate in a survey. This survey aims to help us understand the relationship between mental health and climate change. To participate, you will be asked to answer questions about your demographics, mental health, media consumption, and feelings about climate change.

Purpose & Objectives
The overall goal of this study is to understand mental health in relation to climate change.

Study Procedure
We are asking you to answer a confidential questionnaire. You should complete the questionnaire by yourself. The questionnaire will take approximately 10 minutes. You may skip questions that you do not wish to answer, or stop at any time.

Potential Risks
Some of the questions in this survey ask you to reveal personal information about yourself, your health, and your life experiences, including stressful and traumatic life experiences. Some individuals may experience stress or discomfort while answering these questions. You may skip questions that you do not wish to answer or you may take a short break at any point in the survey. If you do not want to complete the survey, you can simply close the browser window.

The following is a list of services and supports, including phone and online options, if you are in distress or crisis and require some immediate support. If you do not feel like you are in immediate danger, talk to your doctor or another trusted health professional. The supports listed here can be accessed 24 hours a day, 7 days a week, 365 days a year (unless otherwise noted).

Crisis chat: an online crisis support service for adults (is available 24/7 for voice and 4pm to 12am ET for text): www.crisiscentrechat.ca

Kids Help Phone: Free and available 24/7
Call 1-800-668-6868 or text CONNECT to 68686

Potential Benefits
There are no direct benefits to you for participating in this survey. By participating you are helping us better understand how resources can be best used to inform research on climate change and mental health.

Rights and Compensation
This study is completely voluntary. At the end of this survey, you will be automatically directed to a form that will prompt you to enter a draw for a $200 CAD prize, payable by cheque or etransfer from SFU. You must provide your email address to enter. The information you provide in this form will not be linked to your survey responses. If you do not make it to the end of the survey, you will not be able to enter the draw.

Withdrawal from the survey
You may withdraw from the survey by exiting the survey or by skipping all questions and going to the end of the survey to claim your compensation. However, if you decide to quit the survey before finishing it, we will not be able to remove the responses you provided. This is because responses are recorded as each page is completed and we will not know which answers belong to you due to the anonymous nature of the data collection process.

Confidentiality
Your participation is completely voluntary and anonymous. No attempt will be made to identify you based on your survey responses. The data collected may be shared at a later date with other researchers with an interest in validating our findings or conducting additional analyses.

Data collection and storage
This survey is hosted by Survey Monkey, which is U.S. owned. As such, any data you provide will be transmitted and stored in the US (as well as Canada). It is important to remember that privacy laws vary in different countries and may not be as strong as they are in Canada. We encourage you to read the security and privacy policy for the web survey company at the following link: https://www.surveymonkey.com/mp/legal/privacy/

Once the study has concluded, this data will be downloaded to secure servers at Simon Fraser University and deleted from the Survey Monkey platform.

Data Availability
Results from this study will be published in a public report and shared through academic journals and conferences and via our website (www.mhcca.ca) and social media pages. The data you provide will be kept by the Principal Investigators on secure servers at Simon Fraser University. A public version of the data set will also be made available one year after the results of this study are published to allow other researchers to validate our results and explore additional hypotheses.The public version of the data set will not include any information that can identify you (such as the information you may have shared to be entered into the draw).

Contact for information about the study
If you have any questions or need further information with respect to this study, you may contact Dr. Kiffer Card at kcard@sfu.ca.

Contact for concerns about the rights of research subjects
If you have any concerns or complaints about your rights as a research participant and/or your experiences while participating in this study, you may contact the SFU Office of Research Ethics at dore@sfu.ca or 778-782-6618

* 1. **Do you acknowledge and agree to the conditions outlined above?**

- ○ Yes
- ○ No

SFU

Climate Change Impacts Survey

Eligibility Screening

* 2. **What is your age (in Years)?**

- ◯ 15 years or younger
- ◯ 16-24
- ◯ 25-44
- ◯ 45-64
- ◯ 65 years and over

3. **Have you taken this survey before?**

- ◯ Yes
- ◯ No
- ◯ Don't know / Can't remember

Climate Change Impacts Survey

Eligibility Screening part 2

* 4. **Do you live in Canada?**

- ◯ Yes
- ◯ No

Climate Change Impacts Survey

Demographics

5. **What is your highest level of education?**

- ◯ No high school diploma
- ◯ High school diploma or equivalent
- ◯ College, CEGEP or other non-university certificate or diploma
- ◯ Apprenticeship or trades certificate
- ◯ Bachelor's degree
- ◯ Master's degree
- ◯ Professional degree (eg. MD, JD)
- ◯ Doctorate (eg. PhD)

6. **Are you currently a student?**

- ◯ Yes
- ◯ No

7. **Which of these do you identify with?** Check all that apply. The options are listed in alphabetical order.

- ☐ Arab
- ☐ Black
- ☐ Chinese
- ☐ Filipino
- ☐ Indigenous
- ☐ Japanese
- ☐ Korean
- ☐ Latin American
- ☐ South Asian (e.g., East Indian, Pakistani, Sri Lankan, etc.)
- ☐ Southeast Asian (e.g., Vietnamese, Cambodian, Laotian, Thai, etc.)
- ☐ West Asian (e.g., Iranian, Afghan, etc.)
- ☐ White

Another not listed (please specify):

____

8. If you are Indigenous, are you Two-Spirit?

- ◯ I am not Indigenous
- ◯ I am Indigenous and Two-Spirit
- ◯ I am Indigenous, but not Two-Spirit
- ◯ I am Indigenous, but prefer not to say whether I am Two-Spirit

9. **What is your gender identity?** If you have lived experience as trans, a history of gender transition, or are transgender, please select the gender you identify as. We recognize that gender identity questions are imperfect. Please select the option that fits best at this time. The options are listed in alphabetical order.

- ◯ Agender
- ◯ Genderfluid
- ◯ Genderqueer
- ◯ Man
- ◯ Non-binary
- ◯ Trans man
- ◯ Trans woman
- ◯ Woman

I prefer to use another term (please specify):

____

10. **How do you identify sexually?** Check all that apply. The options are listed in alphabetical order.

- ☐ Asexual
- ☐ Bisexual
- ☐ Gay or Lesbian
- ☐ Heteroflexible
- ☐ Pansexual
- ☐ Queer
- ☐ Questioning
- ☐ Straight

I prefer to use another term (please specify):

____

11. **What is your current relationship status?**

- ◯ Single and not dating
- ◯ Single and dating
- ◯ In a relationship

12. **Do you have children?**

- ◯ Yes
- ◯ No, but I plan on having children one day
- ◯ No, and I do not plan on having children
- ◯ No, and I am not sure whether I want to have children

13. **Were you born in Canada?**

- ◯ Yes
- ◯ No, I moved here 5 or more years ago
- ◯ No, I moved here within the past 5 years

14. **Do you identify as a person with a disability?**

- ◯ Yes
- ◯ No

15. **What is your current employment status?** Check all that apply.

- ☐ Employed full time (30+ hours/week)
- ☐ Employed part time (<30 hours/week)
- ☐ Self employed: Professional, Contractor, Business owner
- ☐ On government assistance
- ☐ Student
- ☐ Retired
- ☐ Unemployed
- ☐ Unable to work

16. **What was your total employment income for 2020?**

[ ]

17. **What occupational category do you work in? (Check all that apply)**

- ☐ Management
- ☐ Business, finance and administrative occupations
- ☐ Natural and applied sciences and related occupations
- ☐ Health related occupations
- ☐ Occupations in education, law, and social, community or government services
- ☐ Occupations in art, culture, recreation, and sport
- ☐ Sales and service occupations
- ☐ Trades, transport, and equipment operators, and related occupations
- ☐ Natural resources, agriculture, and related production occupations
- ☐ Occupations in manufacturing and utilities
- ☐ Does not apply to me

18. **How likely or unlikely do you think the industry you're working in will be affected by climate change?**

- ◯ Very unlikely
- ◯ Somewhat unlikely
- ◯ Somewhat likely
- ◯ Very likely
- ◯ Unsure
- ◯ Does not apply to me

19. **If you were beginning your career today, how likely is it that you would choose an alternative career path due to concerns about climate change?**

- ◯ Very likely
- ◯ Somewhat likely
- ◯ Somewhat unlikely
- ◯ Very unlikely
- ◯ Unsure
- ◯ Does not apply to me

20. **How concerned are you that the effects of climate change will impact your ability to access essential resources, such as nutritious food, clean drinking water, safe shelter, and medical services?**

- ◯ Very concerned
- ◯ Somewhat concerned
- ◯ Somewhat not concerned
- ◯ Not concerned at all

SFU

Climate Change Impacts Survey

Environment

21. **What is your postal code?**

Note: We use this data to understand more about the neighbourhood environment in which you live and to link it to weather and climate data.

22. **How many years have you lived at your current residence?**

23. **Which of the options below best describe the community you live in?**

- Urban
- Suburban
- Rural
- Remote

24. **Overall, how satisfied are you with your current housing?**

- Very satisfied
- Satisfied
- Neither satisfied nor dissatisfied
- Dissatisfied
- Very dissatisfied

25. **Overall, how satisfied are you with your current neighbourhood?**

- Very satisfied
- Satisfied
- Neither satisfied nor dissatisfied
- Dissatisfied
- Very dissatisfied

26. **Have you ever been displaced due to a natural disaster or severe changes in your environment?** (e.g., floods, forest fires, drought, loss of agriculture)

- Yes
- No

27. **How likely do you think it is that you will have to relocate to a new town, city, or region due to changes in your environment?** (e.g., floods, forest fires, drought, loss of agriculture)

- Very likely
- Somewhat likely
- Somewhat unlikely
- Very unlikely

28. **Thinking of where you currently live, how likely or unlikely is it that your region will be devastated due to climate related changes?** (e.g., floods, forest fires, drought, loss of agriculture)

- Very likely
- Somewhat likely
- Somewhat unlikely
- Very unlikely
- Don't know or unsure

SFU

Climate Change Impacts Survey

Experiences, Attitudes and Beliefs about Environment

29. **Please rate how often the following statements are true of you.**

	Never	Rarely	Somewhat often	Often	Almost always
Thinking about climate change makes it difficult for me to concentrate.	◯	◯	◯	◯	◯
Thinking about climate change makes it difficult for me to sleep.	◯	◯	◯	◯	◯
I have nightmares about climate change.	◯	◯	◯	◯	◯
I find myself crying because of climate change.	◯	◯	◯	◯	◯
I think, "why can't I handle climate change better?"	◯	◯	◯	◯	◯
I go away by myself and think about why I feel this way about climate change.	◯	◯	◯	◯	◯
I write down my thoughts about climate change and analyze them.	◯	◯	◯	◯	◯
I think, "why do I react this way to climate change?"	◯	◯	◯	◯	◯
My concerns about climate change make it hard for me to have fun with my friends and family.	◯	◯	◯	◯	◯
I have problems balancing my concerns about sustainability with the needs of my family.	◯	◯	◯	◯	◯
My concerns about climate change interfere with my ability to get work or school assignments done.	◯	◯	◯	◯	◯
My concerns about climate change undermine my ability to work to my potential.	◯	◯	◯	◯	◯
My friends say I think about climate change too much.	◯	◯	◯	◯	◯

30. **Please rate how often the following statements are true of you.**

	Never	Rarely	Somewhat often	Often	Almost always
I wish I behaved more sustainably.	◯	◯	◯	◯	◯
I recycle.	◯	◯	◯	◯	◯
I turn off lights.	◯	◯	◯	◯	◯
I try to reduce my behaviours that contribute to climate change.	◯	◯	◯	◯	◯
I feel guilty if I waste energy.	◯	◯	◯	◯	◯
I believe I can do something to address the problem of climate change.	◯	◯	◯	◯	◯

SFU

Climate Change Impacts Survey

General mental health and wellness

31. **Please indicate how often you experienced the following feelings in the past 4 weeks.**

	None of the time	A little of the time	Some of the time	Most of the time	All of the time
Felt nervous.	◯	◯	◯	◯	◯
Felt hopeless.	◯	◯	◯	◯	◯
Felt restless or fidgety.	◯	◯	◯	◯	◯
Felt that everything was an effort.	◯	◯	◯	◯	◯
Felt so sad that nothing could cheer you up.	◯	◯	◯	◯	◯
Felt worthless.	◯	◯	◯	◯	◯
Felt lonely.	◯	◯	◯	◯	◯
Felt like no one understands you.	◯	◯	◯	◯	◯
Felt like you were not a part of your community.	◯	◯	◯	◯	◯
Felt like you had no one to turn to when you needed help.	◯	◯	◯	◯	◯

SFU

Climate Change Impacts Survey

News and social media consumption

32. **Overall, would you say you follow the news:**

- ◯ Very closely
- ◯ Somewhat closely
- ◯ Not very closely
- ◯ Not at all very closely

33. **The United Nations Climate Change Conference, COP26, was held in early-November 2021. How closely did you follow the conference?**

- ◯ Very closely
- ◯ Somewhat closely
- ◯ Not very closely
- ◯ Not at all very closely

34. **As a result of COP26, 197 countries agreed to a new deal, known as the Glasgow Climate Pact, which aims to stave off dangerous climate change. Included in this agreement are commitments to "phase down" use of coal, achieve net-zero emissions in 140 countries, reverse deforestation by 2030, adopt renewable energy sources, promote clean transportation technologies, and provide financial help for climate change mitigation and adaptation.**

How confident are you that the Glasgow Climate Pact will meaningfully prevent continued climate change?

- ◯ Very confident
- ◯ Somewhat confident
- ◯ Not very confident
- ◯ Not confident at all

35. **Where do you get your news about climate change and the environment?** Check all that apply.

- ☐ On social media (e.g., Facebook, Twitter, Reddit, YouTube, Instagram etc..)
- ☐ Television
- ☐ Newspaper or magazine
- ☐ On digital news aggregator (e.g., Google news, Apple News)
- ☐ On the website of a news organization
- ☐ Via email from a family member, friend or colleague
- ☐ None of the above, I don't see news about climate change and the environment

36. **How many times per day do you visit social networking sites (e.g., Facebook, Twitter, Reddit, YouTube, Instagram etc.), on average?**

- ◯ Less than once per day
- ◯ 1-3 times per day
- ◯ 4-8 times per day
- ◯ 9-15 times per day
- ◯ More than 15 times per day

37. **In the past week, on average, how much time PER DAY have you spent actively using social networking sites (e.g., Facebook, Twitter, Reddit, YouTube, Instagram etc.)?**

- ◯ Less than 10 minutes per day
- ◯ 10-30 minutes per day
- ◯ 31-60 minutes per day
- ◯ 1-2 hours per day
- ◯ 2-3 hours per day
- ◯ More than 3 hours per day

38. **When it comes to politics, you would describe yourself as:**

- ◯ Extremely liberal
- ◯ Moderately liberal
- ◯ Slightly liberal
- ◯ Neither liberal nor conservative
- ◯ Slightly conservative
- ◯ Moderately conservative
- ◯ Extremely conservative
